# Plastomes of *Sonchus* (Asteraceae) endemic to the Atlantic Madeira archipelago: Genome structure, comparative analysis, and phylogenetic relationships

**DOI:** 10.1371/journal.pone.0287523

**Published:** 2023-06-22

**Authors:** Hye-Been Kim, Dong-Geol Lee, Seung-Chul Kim

**Affiliations:** 1 Department of Biological Sciences, Sungkyunkwan University, Suwon, Republic of Korea; 2 R&I Center, COSMAX BTI, Pangyo Inno Valley E255, Seongnam, Republic of Korea; Institute for Biological Research, University of Belgrade, SERBIA

## Abstract

The woody *Sonchus* alliance, a spectacular example of adaptive radiation with six genera and approximately 31 species, is found exclusively on three Macaronesian Islands (Madeira, Canaries, and Cape Verdes) in the Atlantic Ocean. Four of the *Sonchus* taxa are restricted to Madeira, including shrubs and small trees at higher elevations (*S*. *fruticosus* and *S*. *pinnatus*), and caudex perennials in the lower coastal areas (*S*. *ustulatus* subsp. *maderensis* and *S*. *ustulatus* subsp. *ustulatus*). The Madeiran *Sonchus* stemmed from a single colonization event that originated from the Canaries < 3 million years ago. However, the plastome evolution and species relationships remains insufficiently explored. We therefore assembled and characterized the plastomes of four *Sonchus* taxa from Madeira and conducted a phylogenomic analysis. We found highly conserved plastome sequences among the taxa, further supporting a single and recent origin. We also found highly conserved plastomes among the cosmopolitan weedy *Sonchus*, Macaronesian *Sonchus* in the Atlantic, and Juan Fernández Islands *Dendroseris* in the Pacific. Furthermore, we identified four mutation hotspot regions (*trn*K-*rps*16, *pet*N-*psb*M, *ndh*F-Ψ*ycf*1, and *ycf*1) and simple sequence repeat motifs. This study strongly supports the monophyly of Madeiran *Sonchus*. However, its relationship with the remaining woody *Sonchus* alliance from the Canary Islands requires further investigation.

## Introduction

Oceanic islands serve as natural laboratories that provide excellent opportunities to investigate the patterns and processes of organismal evolution [1]. The inherent geographical isolation from the continent allows ancestral island populations to become distinct from continental progenitor populations, providing an opportunity to undergo explosive lineage diversification into a wide range of available habitats within the oceanic insular environment—this phenomenon is commonly known as adaptive radiation [2], an example of cladogenetic speciation. Adaptive radiation is often used to explain the diversification of numerous endemic biotas on oceanic islands, providing wonderful opportunities for investigation into speciation [3]. Such cases of evolution include, but are not limited to, well-known examples of Hawaiian *Drosophila* (e.g., *D*. *adiastola* Hardy) [4], Galapagos finches (e.g., *Certhidea olivacea* Gould) [5], and Hawaiian honeycreepers (e.g., *Pinicola enucleator* Linnaeus) [6]. In addition, numerous plant examples, including *Dendroseris* D.Don and *Robinsonia* DC. (e.g., *R*. *gayana* Decne.) on the Juan Fernández Islands, the silversword alliance (e.g., *Dubautia plantaginea* Gaudich.) and *Tetramolopium* Nees (e.g., *T*. *conyzoides* Hillebr.) in Hawaii, and various lineages (e.g., *Echium* L. (e.g., *E*. *candicans* L.f.), *Sonchus* L. (e.g., *S*. *fruticosus* L.f.), and *Argyranthemum* Webb (e.g., *A*. *callichrysum* (Svent.) Humphries) from Macaronesia, reveal the patterns and processes of adaptive radiation on oceanic islands [7–13].

The Sonchinae (currently recognized as Hyoseridinae) is a subtribe from the tribe Cichorieae Lam. & DC. (Asteraceae) distributed globally but with discontinuous habitats in terms of phytogeography, and currently includes 14 genera and approximately 146 species [12]. Several genera of this subtribe, which are mainly found on oceanic islands, exhibit peculiar characteristics compared to their continental species and have been studied as examples of adaptive radiation. Notably, these include *Dendroseris* D.Don in the Juan Fernández Islands, *Thamnoseris* F.Phil. in the San Ambrosio Islands, *S*. *megalocarpa* (Hook.f.) J.M.Black [formerly, *Actites megalocarpa* (Hook.f.) Lander] in Australia, and *Sonchus novae-zelandiae* (Hook.f.) Garn.-Jones [formerly, *Kirkianella novae-zelandiae* (Hook.f.) Allan] and *S*. *grandifolius* Kirk [formerly, *Embergeria grandifolia* (Kirk) Boulos] in New Zealand, as well as the woody *Sonchus* alliance in the Macaronesian Islands. The subgenus *Dendrosonchus*, also known as the woody *Sonchus* alliance, consists of approximately 31 species and is found in Macaronesia, mainly in the Canary Islands, Madeira archipelagos, and Cape Verde. However, *S*. *pinnatifidus* is found on both the Canary Islands and Morocco. They are endemic to the Canary Islands with the exception of five species, among which *S*. *daltonii* is endemic to the Cape Verde, and the remaining four species are endemic to the Madeira archipelagos [14].

**Fig 1:**
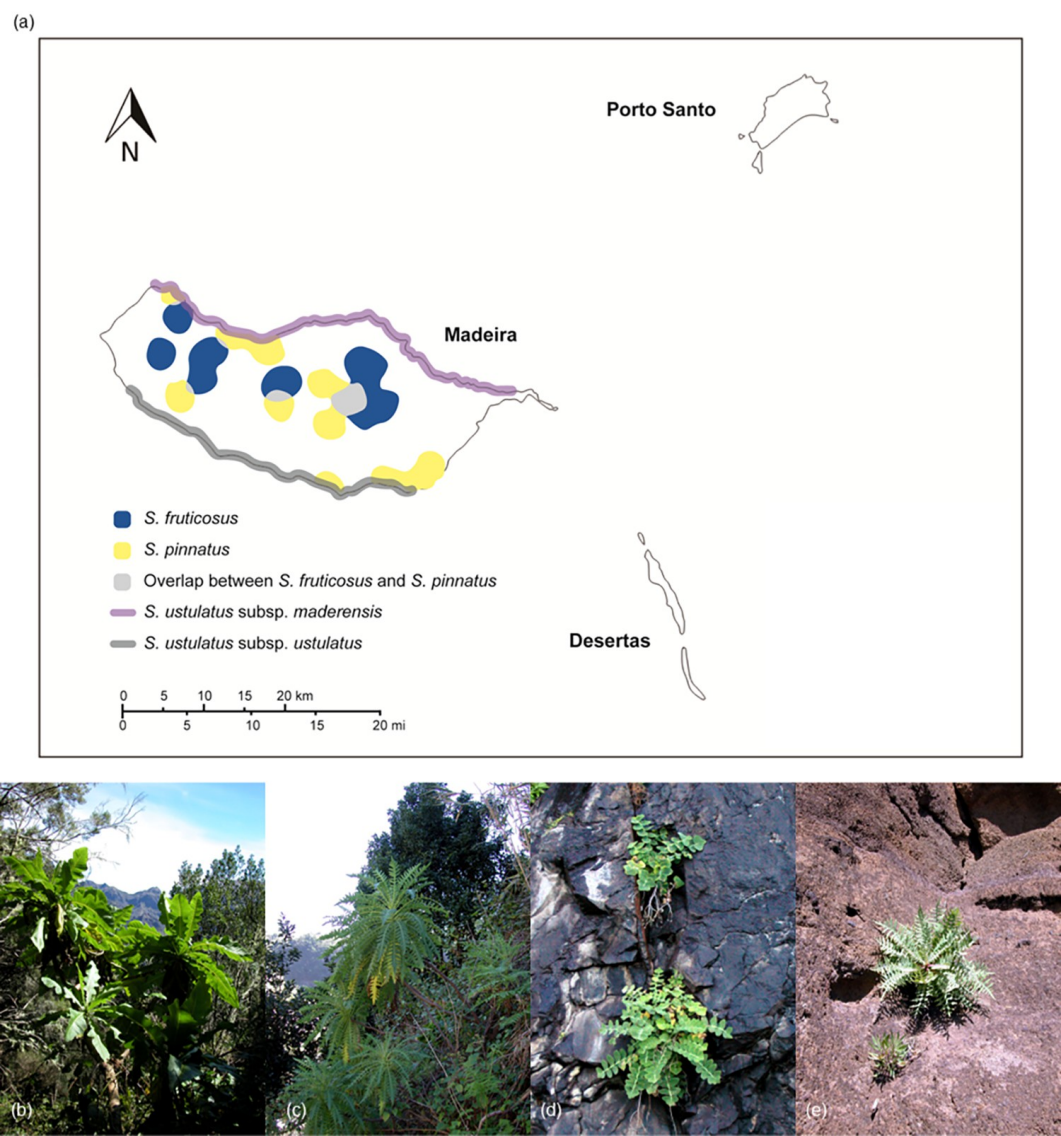
**(a) Map of Madeira archipelago and distribution of the endemic *Sonchus* species to the archipelago.** Occurrence data of *S*. *fruticosus* (purple) and *S*. *pinnatus* (orange) were obtained from the IUCN Red List database (https://www.iucnredlist.org/species/103588658/103588672 and https://www.iucnredlist.org/species/103588709/103588713, respectively). Green areas depict the species overlap areas. The two subspecies of *S*. *ustulatus*, namely subsp. *maderensis* (blue) and subsp. *ustulatus* (red) were based on previous reports [14]. The boundary, which is similar but not identical to the original image and is thus for illustrative purpose only, was obtained from the United States Geological Survey (USGS) National Map Viewer (http://viewer.nationalmap.gov/viewer/). Accessed on May 10, 2023. *Sonchus* species on Madeira (all photos were taken by Seung-Chul Kim); **(b) *S*. *fruticosus*, (c) *S*. *pinnatus*, (d) *S*. *ustulatus* subsp. *maderensis*, and (e) *S*. *ustulatus* subsp. *ustulatus***.

The Madeira archipelago, a part of the floristic region of Macaronesia, is positioned approximately 520 km west of the African country, Morocco, and 400 km north of the Canary Islands. It comprises the main island of Madeira and two smaller eastern islands, Porto Santo and Desertas (Fig 1). Of the 1,226 vascular plants reported in Madeira, 123 (10%) are endemic [15,16]. Four *Sonchus* L. taxa occur exclusively in the Madeira archipelago: *S*. *fruticosus* L.f., *S*. *pinnatus* Aiton, *S*. *ustulatus* subsp. *maderensis* Aldridge, and *S*. *ustulatus* subsp. *ustulatus* Lowe (Fig 1). The Madeira *Sonchus* species form a part of the woody *Sonchus* alliance (ca. 31 species) and originated from the Canary archipelago. They are thought to be monophyletic, as determined by nuclear ribosomal DNA sequences but not yet by chloroplast DNA and represent lineage diversification within the archipelago after inter-archipelago dispersal events [12,14]. These species are categorized into two groups based on distinct life forms: shrub/subshrub types (*S*. *fruticosus* and *S*. *pinnatus*) and caudex perennials (two subspecies of *S*. *ustulatus*) (Fig 1) [15]. *S*. *fruticosus* is a perennial shrub with short, thick branches that reach up to 4 m in height. It has pinnatifid, sinuate, and sessile leaves with triangular lobes and dentate margins. *Sonchus pinnatus* is a perennial shrub that grows up to 2 m in height and has pinnatisect or pinnatipartite and petiolate leaves with lanceolate lobes [15]. The two *S*. *ustulatus* subspecies are herbaceous perennials that reach up to 30 cm height with short sub-woody stems. *Sonchus ustulatus* subsp. *ustulatus* has pinnatipartite to pinnatisect and subsessile leaves, with glaucous on the lower surface and a dentate margin. In contrast, *S*. *ustulatus* subsp. *maderensis* has an entire margin and overlapped leaves with lanceolate terminal lobes. These species occur in different habitats and altitudinal ranges in the Madeira archipelago (Fig 1). *Sonchus fruticosus* is distributed throughout Madeira and Porto Santo, whereas *S*. *pinnatus* and *S*. *ustulatus* subsp. *ustulatus* exclusively occur in Madeira. *Sonchus ustulatus* subsp. *maderensis* is more widespread, occurring on all three islands (Madeira, Porto Santo, and Desertas) [15]. *Sonchus ustulatus* subsp. *ustulatus* and *S*. *ustulatus* subsp. *ustulatus* are commonly distributed in the northern and southern coastal areas of Madeira, respectively. *Sonchus fruticosus* is distributed in Laurisilva and the moist ravines in the interior of Madeira, at altitudes of 800–1200 m, while *S*. *pinnatus* commonly is distributed on rocky slopes at 1000–1400 m and lower altitudes [15].

The advent of next generation sequencing (NGS) allowed for the rapid assembly and characterization of whole plastid genome sequences in numerous land plant lineages. These plastome resources have proven useful in resolving difficult and obscure phylogenetic relationships and developing efficient plastid markers for DNA barcoding and phylogeographic and phylogenetic studies [17]. In an ongoing effort to establish robust and highly resolved plastome-based phylogenetic relationships between *Sonchus* and related genera in the subtribe Hyoseridinae, we characterized several herbaceous and woody *Sonchus* species [18–22]. In particular, the plastomes of the woody *Sonchus* alliance have been characterized with special emphasis on species from the Canaries [18,21]. However, information on the plastome organization and variations among the four Madeiran taxa and their relationships with the species in the Canaries remains limited. Definitive evidence for the monophyly of Madeira *Sonchus* based on cpDNA is also lacking. Therefore, this study aimed to: (1) determine the complete plastomes of four Madeira-endemic *Sonchus* taxa; (2) compare their gene content, order, and any changes in organization; (3) conduct comparative genomic analyses to identify highly variable hotspot regions and chloroplast simple sequence repeat (cpSSR) markers; and (4) elucidate the phylogenetic relationships among the four Madeira *Sonchus* taxa and assess their relationships with the Canary Islands congeneric taxa.

## Methods and methods

### Material preparation, DNA extraction, genome sequencing, and annotation

One the 2002 expedition to Madeira, four endemic *Sonchus* taxa were collected freshly from the island (see voucher and collection information in [14]). The total genomic DNA of three taxa, *S*. *pinnatus*, *S*. *ustulatus* subsp. *ustulatus*, and *S*. *ustulatus* subsp. *maderensis* was isolated using the Exgene™ Plant SV mini kit (GeneAll, Seoul, Korea). *Sonchus fruticosus*’s DNA was extracted using the CTAB method [23]. The concentration and quality of the extracted DNA were verified using 1% agarose gel electrophoresis. Next-generation sequencing (NGS) was performed at Macrogen Corporation (Seoul, Korea), and approximately six gigabytes of raw NGS sequence data was generated for each taxon. As shown in Table 1, the depth of coverage of 500X or higher indicates that sufficient sequencing was conducted for chloroplast genome assembly.

**Table 1:** Summary of the complete chloroplast genome of the Madeira endemic *Sonchus*.

Species	*S*. *fruticosus*	*S*. *pinnatus*	*S*. *ustulatus* subsp. *maderensis*	*S*. *ustulatus* subsp. *ustulatus*
GenBank accession number	MN537816	MN542414	MN537815	MN537814
Total cpDNA^a^ size (bp)	152,410	152,410	152,426	152,411
LSC size (bp)	84,324	84,324	84,335	84,325
SSC size (bp)	18,592	18,592	18,599	18,592
IR size (bp)	24,747	24,747	24,746	24,747
Number of different genes	131	131	131	131
Number of different protein-coding genes	87	87	87	87
Number of different tRNA genes	37	37	37	37
Number of different rRNA genes	6	6	6	6
GC content (%)	37.6	37.6	37.6	37.6
Depth of coverage	2770	537	2063	1875
Habitat and Island	Rocky areas (e. g. inland cliffs, mountain peaks), Forest; M, PS	Rocky areas (e. g. inland cliffs, mountain peaks); M	Moist rocky and shady areas (North coast); M, PS, D	Dry rocky and sunny areas (South coast); M
Height (m)	4	2	0.3	0.3
Altitude (m)	800–1200	1000–1400 (it is also found at lower altitude)	100–300	100–300

^a^cpDNA, chloroplast DNA; LSC, large single-copy region; SSC, small single-copy region; IR, inverted repeat; GC, guanine-cytosine; bp, base pairs; cpDNA, chloroplast DNA; M, Madeira Island; PS, Porto Santo; D, Desertas Islands.

An Illumina paired-end (PE) genomic library was constructed and sequenced using the Illumina HiSeq platform, according to the standard Illumina PE protocol in the TruSeq Nano DNA Sample Preparation Guide. The sequence reads were assembled using the *de novo* genomic assembler Velvet 1.2.10 [24]. In the case of *S*. *pinnatus*, we performed PCR confirmation for three ambiguous regions (two in the large single copy, LSC, and one in the small single copy, SSC) to assemble the circular plastid genome. PCR gap filling was performed using the Inclone™ Taq DNA polymerase kit (IncloneBiotech Co., Yongin, Korea), with a final volume of 50 μL. The PCR product was purified using the Inclone™ Gel & PCR Purification Kit (Inclonebiotech Co., Yongin, Korea) and sequenced at Macrogen Corporation (Seoul, Korea).

Protein-coding regions (CDS) were annotated using CPGAVAS2 [25], and tRNAs were predicted using ARAGORN v1.2.38 (http://mbio-serv2.mbioekol.lu.se/ARAGORN/; [26]) and tRNAscan-SE (http://lowelab.ucsc.edu/tRNAscan-SE/; [27]). RNAmmer 1.2 Server [28] was used to annotate rRNAs. These were annotated and corrected based on four congeneric taxa from the Canary Islands, namely *S*. *canariensis* (NC042381), *S*. *acaulis* (NC042382), *S*. *webbii* (NC042383), and *S*. *leptocephalus* (MN334533) using Geneious Prime 2020.0.3 (Biomatters Ltd., Auckland, New Zealand). Each circular genome map was visualized using OGDRAW v. 1.3.1 (http://ogdraw.mpimp-golm.mpg.de/; [29]) and MPI-MP CHLOROBOX online. Complete cp genome sequences were deposited in GenBank (MN537814, MN537815, MN537816, and MN542414).

### Identification of highly divergent regions

We estimated the nucleotide diversity of woody *Sonchus* species and analyzed the DNA polymorphisms using DnaSP v. 6 [30]. This software computed the sliding window for nucleotide diversity (*Pi*) with a 100 bp window length and a 25 bp step size. We executed the LAGAN alignment mode using the mVISTA program (http://genome.lbl.gov/vista/mvista/; [31,32]) to align the four Madeira *Sonchus* taxa. *Sonchus fruticosus* was used as the reference. Four plastid genomes were aligned using MAFFT v. 7 (https://mafft.cbrc.jp/alignment/software/; [33]), and gene orientation was checked using Geneious software. The window size and resolution were 100 bp and 60 bp, respectively.

### Repeat sequence analysis

MIcroSAtellite identification software (MISA; http://pgrc.ipk-gatersleben.de/misa/) was used to detect simple sequence repeat (SSR) motifs with repeat units ranging from one to six nucleotides [34]. The minimum number of nucleotide repeats was set to 15 for mononucleotide repeats (1–15), five for dinucleotide (5–3) repeats, and three for trinucleotide (3–3), tetranucleotide (4–3), pentanucleotide (5–3), and hexanucleotide (6–3) repeats.

### Codon usage bias, RNA editing site, and genes under positive selection

The codon usage bias was calculated using MEGA-X [35]. We compared the codon distribution and relative synonymous codon usage (RSCU) values of the study group. Moreover, we conducted RNA-editing on Madeiran *Sonchus* species using PREP-Cp (http://prep.unl.edu/; [36]), with default settings (cutoff value of 0.8). Each Madeira *Sonchus* had 87 protein-coding sequences (CDSs). Thirty-five of the total CDSs were analyzed as 52 plastid genes were not supported by the PREP-Cp. We calculated the Ka/Ks ratio of each pair in Madeira *Sonchus* using DnaSP v. 6 [30] to evaluate selective pressure.

### Phylogenetic analysis

To determine the phylogenetic position and relationships of four Madeiran *Sonchus* species, we selected a total of 18 complete chloroplast genomes of the subtribe Hyoseridinae (formerly known as Sonchinae). *Reichardia ligulata* (MN893255), which diverged early in Hyoseridinae, was used as the outgroup. We selected herbaceous *Sonchus* spp., including *S*. *boulosii* (NC042244), *S*. *arvensis* (NC054161), *S*. *oleraceus* (MK371006), *S*. *asper* (MK371015), and *S*. *brachyotus* (MT850047). Moreover, we included five previously sequenced the woody *Sonchus* alliance taxa from the Canary Islands, namely *S*. *bupleuroides* (MK371017), *S*. *webbii* (NC042383), *S*. *leptocephalus* (MN334533), *S*. *acaulis* (MK033507), and *S*. *canariensis* (MK033506). Finally, we included seven *Dendroseris* species, which are endemic to the Juan Fernández Islands in the Pacific Ocean and belong to the Hyoseridinae, namely *D*. *berteroana* (NC051923), *D*. *pinnata* (NC051926), *D*. *marginata* (NC051925), *D*. *litoralis* (NC051922), *D*. *macrantha* (NC051924), *D*. *micrantha* (NC051921), and *D*. *pruinata* (NC051920). We aligned the chloroplast genomes using MAFFT v. 7 with default settings and edited them manually using Geneious software. A maximum-likelihood (ML) tree and a Bayesian tree were inferred using IQ-TREE v. 1. 6.12 [37], and MrBayes v.3.2.7 [38], respectively, with partitioning the alignment by the gene annotations. The best-fit substitution model for each partition was chosen according to the Bayesian information criterion (BIC) score and weight, using ModelFinder [39] implemented in IQ-TREE. The branch bootstrap support (BS) of ML tree was calculated with 1000 bootstrap replicates, and for Bayesian inference, the Markov chain Monte Carlo (MCMC) was run for four chains for 2,000,000 generations with sampling a tree each 100 generations and burning out the first 25% of the sampled trees. We also conducted a maximum parsimony (MP) analysis using PAUP version 4.0a [40]. The default heuristic search options included starting trees via stepwise simple sequence addition with one tree held at each step, the tree-bisection-reconnection (TBR) branch-swapping algorithm, steepest descent, and MulTrees option in effect, zero branch length collapsed, and topological constraints not enforced. BS was calculated from 1000 replicates using the same heuristic search options to evaluate the robustness of the groups.

## Results

### Comparative genome analysis of four *Sonchus* taxa according to gene content, order, and organization

**Fig 2:**
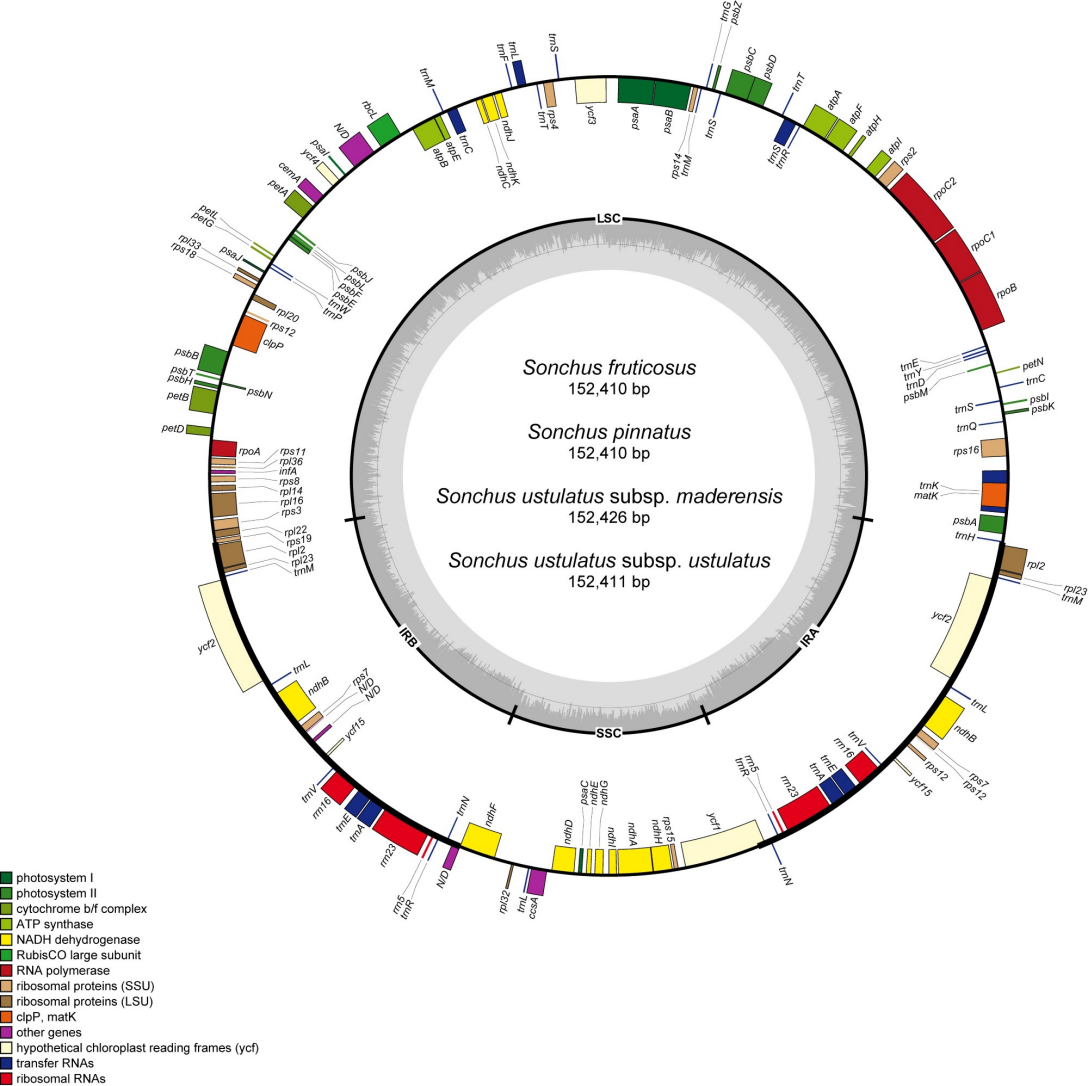
Gene map of Madeira endemic *Sonchus* created using OGDRAW. The genes inside and outside the circle are transcribed in clockwise and counterclockwise directions, respectively. Genes belonging to different functional groups are shown in different colors. Thick lines indicate the extent of the inverted repeats (IRa and IRb) that separate the genomes into large single copy (LSC) and small single copy (SSC) regions.

**Fig 3:**
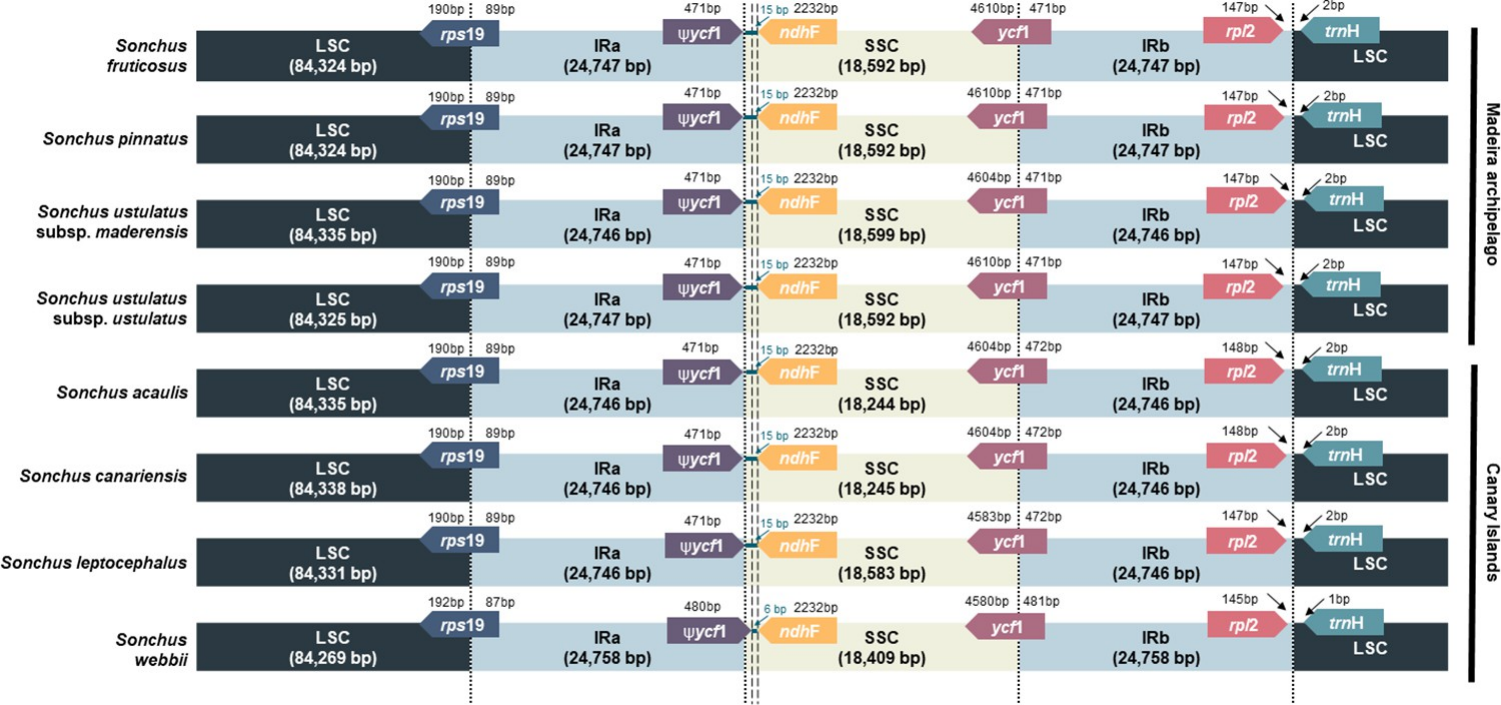
Comparisons of the border positions of the LSC, SSC, and IR regions among eight plastomes of the woody *Sonchus* alliance from Madeira (*S*. *fruticosus*, *S*. *pinnatus*, *S*. *ustulatus* subsp. *maderensis*, and *S*. *ustulatus* subsp. *ustulatus*) and the Canary Islands (*S*. *acaulis*, *S*. *canariensis*, *S*. *leptocephalus*, and *S*. *webbii*). Ψ indicates pseudogenes. Gene names are indicated in boxes and their lengths in the corresponding regions are displayed above the boxes.

Each Madeira chloroplast genome was composed of 131 CDSs, 87 genes, 37 tRNAs, and six rRNAs (Table 1). The total cp DNA size among the four taxa ranged from 152,410 bp (*S*. *fruticosus* and *S*. *pinnatus*), 152,411 bp (*S*. *ustulatus* subsp. *ustulatus*) to 152,426 bp (*S*. *ustulatus* subsp. *maderensis*) (Fig 2), which are assembled with high depths of coverage, ranged from 537 (*S*. *pinnatus*) to 2770 (*S*. *fruticosus*). The sizes of the LSC, SSC, and IR were nearly identical among the four plastomes (Table 2). In particular, two morphologically distinct shrubby species, *S*. *fruticosus* and *S*. *pinnatus*, presented identical chloroplast genome sizes with nearly identical sequences, except for three point-mutations on each *clp*P intron, and the *trn*K-*rps*16 and *psa*A-*ycf*3 intergenic spacers. The four Madeira taxa had identical gene compositions (Table 1). Seven genes (*pet*B, *atp*F, *ndh*A, *rpl*2, *rpl*16, *rps*16, and *rpo*C1) contained a single intron and three genes (*clp*P, *rps*12, and *ycf*3) contained two introns (Table 2). We found little variation for the border positions of the LSC, SSC, and IR regions among the chloroplast genomes of *Sonchus* species in Madeira and the Canary Islands (Fig 3). The Madeira *Sonchus* showed a difference in the *ycf*1 region between SSC and IRb in *S*. *ustulatus* subsp. *maderensis*, compared with the remaining taxa. For the Canary Islands *Sonchus* plastomes, *S*. *webbii* contained a 6 bp gap between ψ*ycf*1 and *ndh*F, while others contained a 15 bp gap at the SSC/IRa. The ψ*ycf*1 gene of *S*. *webbii* (480 bp) was slightly longer than the others (471 bp).

**Table 2:** Genes present in the complete chloroplast genome of Madeira endemic *Sonchus*.

Category	Gene name
Photosystem Ⅰ	*psa*A, *psa*B, *psa*C, *psa*I, *psa*J, *ycf* 3^b^, *ycf* 4
Photosystem Ⅱ	*psb*A, *psb*B, *psb*C, *psb*D, *psb*E, *psb*F, *psb*H, *psb*I, *psb*J, *psb*K, *psb*L, *psb*M, *psb*N, *psb*T, *psb*Z
Cytochrome b6/f complex	*pet*A, *pet*B^a^, *pet*D, *pet*G, *pet*L, *pet*N
Cytochrome C synthesis	*ccs*A
ATP synthase	*atp*A, *atp*B, *atp*E, *atp*F^a^, *atp*H, *atp*I
RuBisCO	*rbc*L
NADH oxidoreductase	*ndh*A^a^, *ndh*B^a^^,^^c^, *ndh*C, *ndh*D, *ndh*E, *ndh*F, *ndh*G, *ndh*H, *ndh*I, *ndh*J, *ndh*K
Large subunit ribosomal proteins	*rpl*2^a^^,^^c^, *rpl*14, *rpl*16^a^, *rpl*20, *rpl*22, *rpl*23^c^, *rpl*32, *rpl*33, *rpl*36
Small subunit ribosomal proteins	*rps*2, *rps*3, *rps*4, *rps*7^c^, *rps*8, *rps*11, *rps*12^b^^,^^c^^,^^d^, *rps*14, *rps*15, *rps*16^a^, *rps*18, *rps*19
RNA polymerase	*rpo*A, *rpo*B, *rpo*C1^a^, *rpo*C2
Translation initiation factor	*inf*A
Others	*acc*D, *cem*A, *clp*P^b^, *mat*K
Unknown function genes (Conserved reading frames)	*ycf* 1^c^, *ycf* 2^c^, *ycf* 15^c^
Ribosomal RNAs	*rrn*5^c^, *rrn*16^c^, *rrn*23^c^
Transfer RNAs	*trn*A-UGC^a^^,^^c^, *trn*C-ACA^a^, *trn*C-GCA, *trn*D-GUC, *trn*E-UUC^a^^,^^c^, *trn*E-UUC, *trn*F-GAA, *trn*G-GCC, *trn*G-UCC^a^, *trn*H-GUG, *trn*K-UUU^a^, *trn*L-CAA^c^, *trn*L-UAA, *trn*L-UAG, *trn*M-CAU^c^, *trn*M-CAU, *trn*M-CAU, *trn*N-GUU^c^, *trn*P-UGG, *trn*Q-UUG, *trn*R-CCA^c^, *trn*R-UCU, *trn*S-GGA, *trn*S-UGA, *trn*S-GCU, *trn*T-UGU, *trn*T-GGU, *trn*V-GAC^c^, *trn*W-CCA, *trn*Y-GUA

^a^ Genes containing a single intron

^b^ Genes containing two introns

^c^ Two gene copies in the IRs

^d^ Trans-splicing gene.

### Simple sequence repeats, codon usage bias, and RNA editing sites

**Fig 4:**
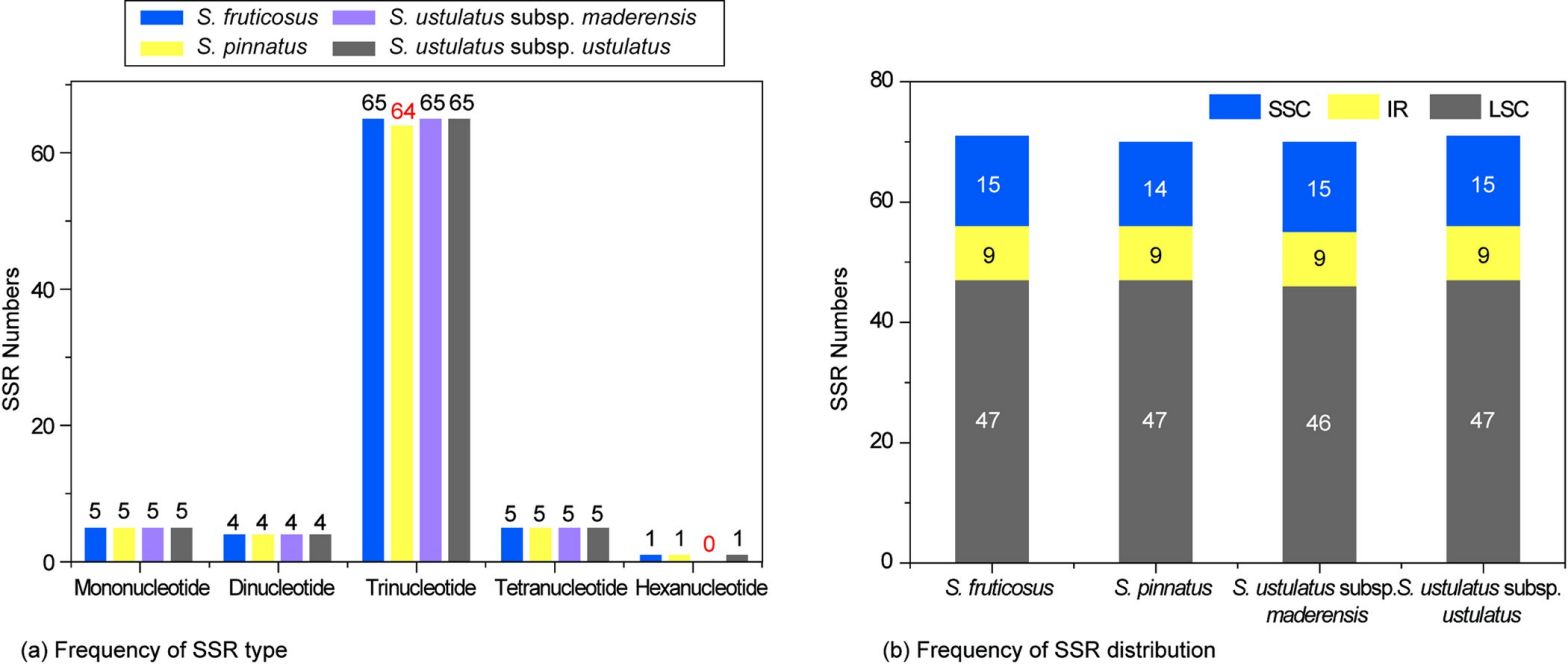
The simple sequence repeat number per distribution and repeat type of four Madeira endemic *Sonchus*. (a) Frequency of SSR type and (b) Frequency of SSR distribution in the chloroplast genome.

We investigated SSRs among the four Madeira *Sonchus* plastomes and found a similar total number of SSRs (Fig 4). A total of 71 SSRs were found in *S*. *fruticosus* and *S*. *ustulatus* subsp. *ustulatus*, and 70 in *S*. *pinnatus* and *S*. *ustulatus* subsp. *maderensis* (Fig 4A). The most SSRs were detected in the coding regions (53%), and the most frequent SSR type was trinucleotides (81%) (Fig 4A). The LSC region contained the most SSRs (46–47 SSRs; 58–59%), compared to the SSC (15 SSRs; 21%) and IR (9 SSRs; 12.8%) regions (Fig 4B). All four plastomes had an equal number of mononucleotide, dinucleotide, and tetranucleotide repeats, but different numbers of trinucleotide and hexanucleotide repeats (Fig 4A). In terms of trinucleotide motifs, *S*. *pinnatus* contained 64 repeats, whereas the remaining taxa contained 65 repeats. Additionally, *S*. *ustulatus* subsp. *maderensis* did not contain a hexanucleotide repeat, whereas the remaining species had one hexanucleotide repeat. Interestingly, we found identical frequencies of SSR distribution and types between the two morphologically divergent *S*. *fruticosus* and *S*. *ustulatus* subsp. *ustulatus*.

**Fig 5:**
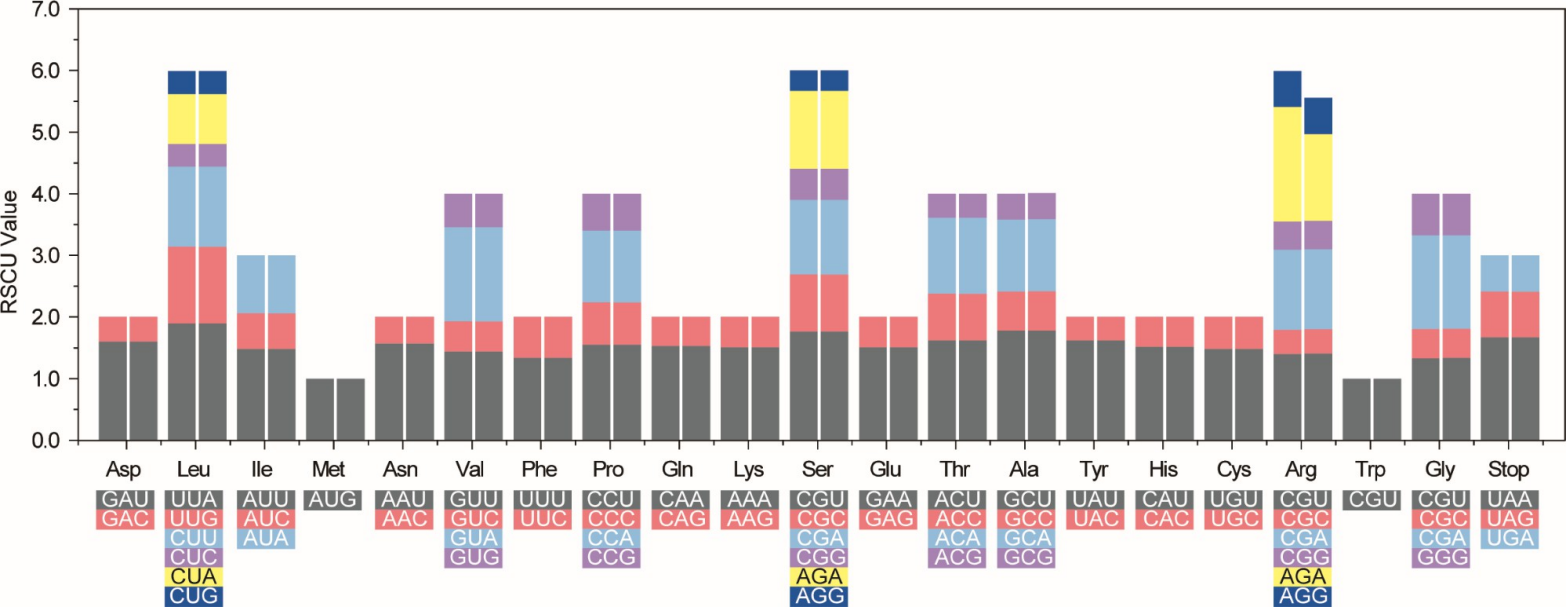
Codon usage bias in protein-coding genes of Madeira endemic *Sonchus*. RSCU = relative synonymous codon usage (Left bar: *S*. *fruticosus*, *S*. *pinnatus* and *S*. *ustulatus* subsp. *ustulatus*; Right bar: *S*. *ustulatus* subsp. *maderensis*).

Analysis of relative synonymous codon usage (RSCU) based on the protein-coding genes revealed an average codon usage ranging from 22,774 (*S*. *ustulatus* subsp. *maderensis*) to 22,776 (*S*. *fruticosus*, *S*. *pinnatus*, and *S*. *ustulatus* subsp. *ustulatus*) among the four Madeira *Sonchus* taxa, with consistent patterns of frequently used codons among them (S1 Table). The highest RSCU value was observed with the UUA codon used for Leucine (1.9), followed by that of AGA for Arginine (1.41–1.86) and GCU for Alanine (1.78) (Fig 5). The lowest RSCU value was observed with the AGC codon used for serine (0.33), and CUG and CUC for leucine (0.37).

We also predicted RNA editing sites in the Madeira *Sonchus* species. All species had 48 RNA editing sites with the same cutoff value of 0.8 (S2 Table). These editing sites were present in 20 of the 35 protein-coding genes. Nine RNA editing sites were found in *ndh*B, five in *ndh*D, four in *acc*D and *rpo*C1, three in *mat*K and *ndh*A, and two in *atp*A, *ccs*A, *ndh*G, *pet*B, *rpo*C2, and *rps*14. One editing site was identified for each *atp*I, *ndh*F, *psb*F, *psb*L, *rpl*20, *rpo*A, *rpo*B, and *rps*2. All nucleotide changes were cytosine (C) to thymine (T) transitions. The most frequent transitions were conversions from proline (P) to leucine (L).

### Sequence divergence and mutation hotspot regions

**Fig 6:**
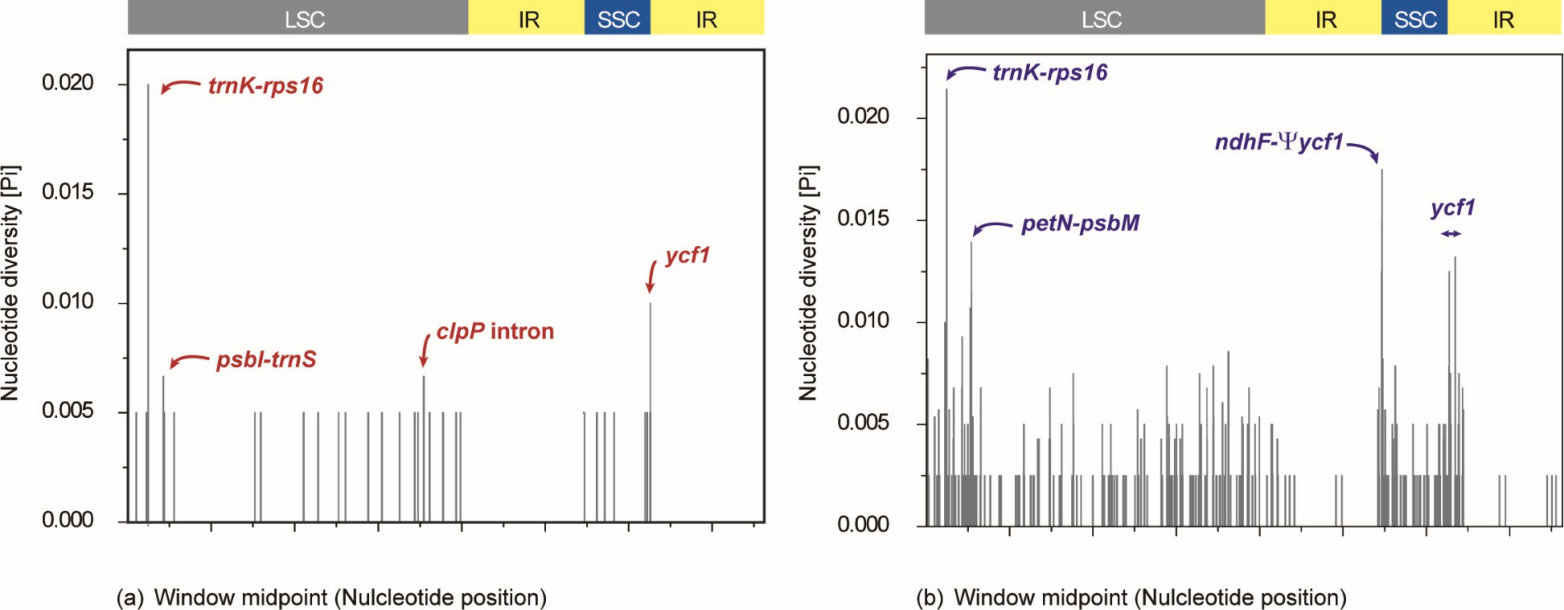
(a) Nucleotide diversity (Pi) of the four Madeira *Sonchus* plastomes and (b) eight Macaronesia the woody *Sonchus* alliance plastomes. The four most divergent regions are suggested as divergence hotspots based on Pi ≥ 0.005 for Madeira *Sonchus*, and Pi ≥ 0.01 for Macaronesia *Sonchus* species.

We calculated the nucleotide diversity of eight woody *Sonchus* alliances on the Macaronesian Islands (i.e., Madeira and the Canary Islands) using DnaSP (Fig 6A and 6B). The average nucleotide diversity (Pi) was 0.0005 and ranged from 0 to 0.015. The SSC region had the highest nucleotide diversity (Pi = 0.00117), whereas each of the two IR regions had the lowest (P = 0.0001). The LSC region presented nucleotide diversity of 0.00058. For the eight Macaronesia *Sonchus* plastomes, we found four mutation hotspots with Pi values of ≥ 0.01, including two intergenic regions (*trn*K-*rps*16 and *pet*N-*psb*M) in the LSC, and one protein-coding (*ycf*1) and one intergenic region (*ndh*F-ψ*ycf*1) in the SSC (Fig 6B). We also found four highly variable regions (i.e., *trn*K-*rps*16 and *psb*I-*trn*S, *clp*P intron, and *ycf*1) when only Madeiran species plastomes were considered (Fig 6A).

**Fig 7:**
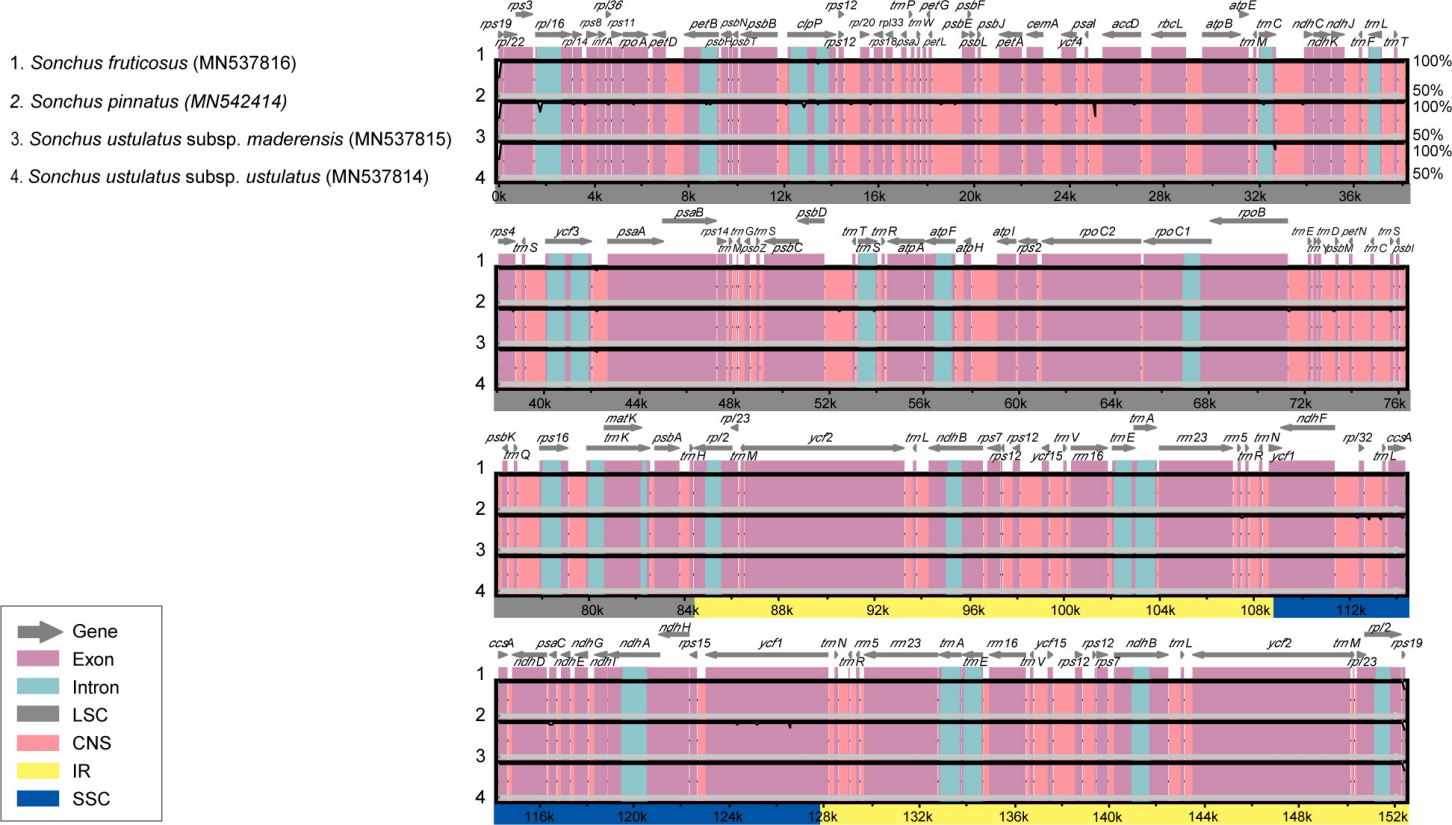
Alignment visualization of chloroplast genome sequences among Madeira endemic *Sonchus*. VISTA-based identity plots showed the sequence identity of three chloroplast genomes with *S*. *fruticosus* as reference. The vertical scale indicates the percent identity from 50% to 100%. The horizontal scale indicates the genome base positions. Gray arrows above the alignment indicate the position and direction of each gene.

The mVISTA plots, with *S*. *fruticosus* as a reference, showed a high degree of synteny and gene order conservation among three of the Madeira *Sonchus* plastomes (Fig 7). We found 56 polymorphic sites. Not surprisingly, the intergenic spacer region (IGS) contained 55.4% of the total mutations, while the exon and intron regions contained 23.2% and 21.4%, respectively (Table 3). Approximately 55% of the polymorphisms were base substitutions, 29% were transitions (A/G or C/T), and 71% were transversions (A/C, A/T, C/G, and G/T). Of the 25 polymorphic indels (insertions and deletions), 10 (40%) were non-SSR indels, and the remaining 15 (60%) were poly A/T/G SSR indels. The longest non-SSR indels at 17 bp were located in *the rps*16-*trn*K and *rpl*16 introns. The most divergent regions included the *rps*16 intron, *clp*P intron, *psa*I-*acc*D, *atp*F-*atp*H, *rps*16-*trn*K, *rpl*32-*trn*L, and *ycf*1 (Fig 7). The most variable region between the four Madeira *Sonchus* plastomes was the LSC (75.0%) region, followed by the SSC (23.2%) and IR (1.7%) regions.

**Table 3:** Distribution of base substitutions and insertion/deletions (indels) in the chloroplast genomes of four Madeira *Sonchus* species.

No.	Location	Region	*S*. *fruticosus*	*S*. *pinnatus*	*S*. *ustulatus* subsp. *maderensis*	*S*. *ustulatus* subsp. *ustulatus*
1	*rpl*16	Intron	TATTATTTATTAA	TATTATTTATTAA	-	TATTATTTATTAA
2	*rpl*16	Intron	-	-	TCTGAATT	-
3	*rpl*16*-rpl*14	IGS*	A	A	-	A
4	*rpl*14*-rps*8	IGS	-	-	A	-
5	*rpl*36	Exon	G	G	T	G
6	*rpo*A	Exon	C	C	T	C
7	*pet*B	Intron	A	A	G	A
8	*pet*B	Intron	G	G	C	G
9	*psb*B*-clp*P	IGS	C	C	A	C
10	*clp*P	Intron	TAAGGA	TAAGGA	-	TAAGGA
11	*clp*P	Intron	A	G	G	A
12	*clp*P	Intron	-	-	A	-
13	*clp*P	Intron	-	-	A	-
14	*rps*12*-rpl*20	IGS	G	G	A	G
15	*rpl*20*-rps*18	IGS	A	A	T	A
16	*pet*L*-psb*E	IGS	-	-	T	-
17	*pet*L*-psb*E	IGS	-	-	T	-
18	*pet*L*-psb*E	IGS	A	A	C	A
19	*cem*A*-ycf*4	IGS	C	C	A	C
20	*psa*I-*acc*D	IGS	-	-	TAATTAGATTCTATTTA	-
21	*acc*D	Exon	G	G	C	G
22	*trn*C	Intron	C	C	A	C
23	*trn*C*-ndh*C	IGS	-	-	-	GCCCTATA
24	*trn*C*-ndh*C	IGS	A	A	C	A
25	*rps*4	Exon	C	C	T	C
26	*ycf*3*-psa*A	IGS	T	G	G	G
27	*ycf*3*-psa*A	IGS	A	A	-	A
28	*psb*D*-trn*T	IGS	C	C	T	C
29	*trn*G	Intron	C	C	A	C
30	*atp*F-*atp*H	IGS	AATATTA	AATATTA	-	-
31	*rpo*B*-trn*E	IGS	A	A	-	A
32	*rpo*B*-trn*E	IGS	T	T	-	T
33	*trn*D*-psb*M	IGS	T	T	G	T
34	*psb*M*-pet*N	IGS	-	-	G	-
35	*pet*N*-trn*C	IGS	A	A	-	A
36	*trm*C*-trm*S	IGS	C	C	T	C
37	*trn*S-*psb*I	IGS	T	T	G	G
38	*rps*16	Intron	G	G	-	G
39	*rps*16*-trn*K	IGS	-	-	GCAGTGCCCATCCAACA	-
40	*rps16-trnK*	IGS	T	T	-	T
41	*rps*16*-trn*K	IGS	T	G	G	G
42	*trn*K	Intron	C	C	A	C
43	*rrn*5*-trn*R	IGS	A	A	-	A
44	*ndh*F	Exon	G	G	A	G
45	*ndh*F	Exon	T	T	G	T
46	*ndh*F*-rpl*32	IGS	G	G	T	G
47	*rpl*32*-trn*L	IGS	-	-	TCGACCT	-
48	*rpl*32*-trn*L	IGS	-	-	TAAGA	-
49	*ccs*A	Exon	T	T	A	T
50	*ndh*D*-psa*C	IGS	-	-	A	-
51	*psa*C	Exon	G	G	A	G
52	*ycf*1	Exon	G	G	T	G
53	*ycf*1	Exon	C	C	A	C
54	*ycf*1	Exon	T	T	A	T
55	*ycf*1	Exon	C	C	G	C
56	*ycf*1	Exon	TTATTC	TTATTC	-	TTATTC

^a^IGS: Intergenic spacer.

### Monophyly and species relationships in Madeira archipelago

**Fig 8:**
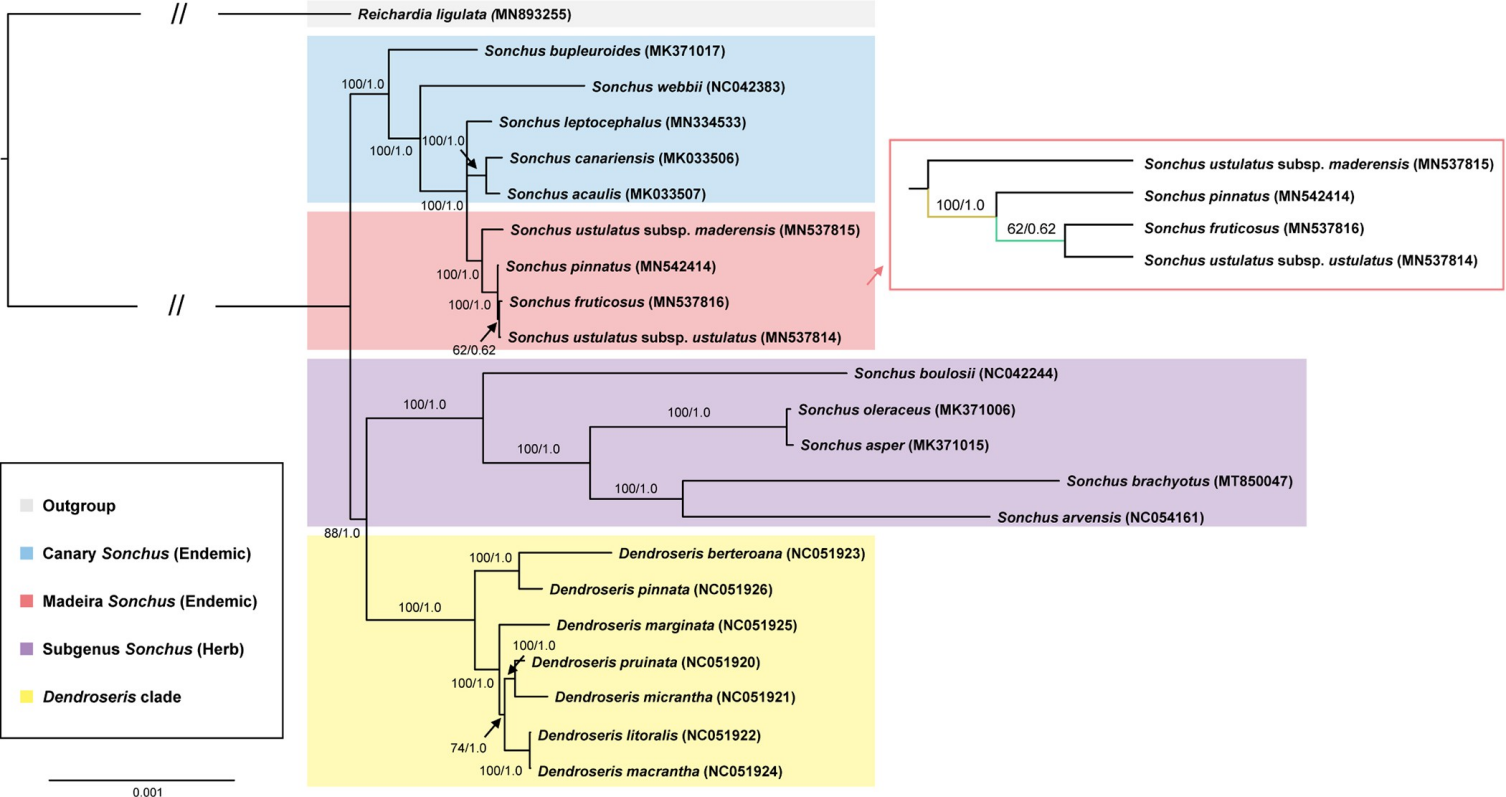
Maximum-likelihood (ML) tree generated by IQ-TREE, based on 18 species of subtribe Hyoseridinae. *Reichardia ligulata* was used as an outgroup based on previous studies [12,41]. The numbers above the nodes present the bootstrap values of 1000 replicates and posterior probability from the Bayesian inference.

The topologies of the ML tree and the Bayesian inference (BI) tree were identical, and both strongly suggested the monophyly of the woody *Sonchus* alliance in the Macaronesian Islands (100% BS and 1.0 posterior probability (PP)) (Fig 8). The MP tree (not shown) also strongly supported the monophyly of *Sonchus* and its relationships with other representative genera of the subtribe Hyoseridinae. It is highly likely that the woody *Sonchus* alliance from the Macaronesian Islands was derived from a common ancestor. Also, the Madeira *Sonchus* formed a monophyletic clade based on the complete plastome sequences (100% BS). The two subspecies of *S*. *ustulatus*, namely subsp. *ustulatus* and subsp. *maderensis*, are not in a monophyletic clade. Rather, subsp. *ustulatus* shares a more recent common ancestor with *S*. *pinnatus* and *S*. *fruticosus* than with conspecific subsp. *maderensis* (Fig 8).

## Discussion

### Madeiran *Sonchus* chloroplast genomes

We found highly conserved plastomes based on their size, gene content, order, and organization between the four Madeira *Sonchus*. The Madeira *Sonchus* species (152,410 bp—152,426 bp) had slightly larger plastomes than the weedy *Sonchus* (151,849 bp -151,967 bp) and the Canary Islands *Sonchus* species (152,071 bp—152,406 bp). However, the gene content and organization were highly conserved among the groups [20,21]. The woody *Sonchus* alliance from Madeira and the Canaries had slightly larger plastomes than the continental herbaceous congeneric species. In addition, very similar plastome sizes (152,199 bp—152,619 bp for *Dendroseris* and 152,071 bp—152,406 bp for the woody *Sonchus* alliance), gene content, and organization was observed between the Atlantic Ocean diploid *Sonchus* endemics and the Pacific Ocean Juan Fernández Islands tetraploid *Dendroseris*, which belongs to the same subtribe Hyoseridinae as *Sonchus* [42]. This indicates that, even though these insular endemics from the Pacific and Atlantic Oceans are morphologically and ecologically divergent, they have highly conserved plastomes that suggest their recent independent origins.

Using the same SSR search parameters (1–15, 2–5, 3–3, 4–3, 5–3, and 6–3) as the weedy *Sonchus* (*S*. *asper*, *S*. *oleraceus*; [20]) and the woody *Sonchus* alliance in the Canaries (*S*. *canariensis*, *S*. *acaulis*, *S*. *webbii*; [21]), slightly fewer SSRs were found among the Madeira *Sonchus*: 71 versus 79 for the weedy *Sonchus* and 80 for *S*. *acaulis*, 78 for *S*. *canariensis*, and 78 for *S*. *webbii*. Most of the trinucleotide motifs found in the Madeira *Sonchus* plastomes coincided with the weedy and Canary Islands *Sonchus* species [20,21]. We found a similar number of cpSSRs (71–74) and trinucleotide repeats with the highest frequency (87%) in the Juan Fernández Island endemic *Dendroseris* [42]. However, the LSC region contained the most SSRs (62%) in *Dendroseris*. The cpSSR markers identified in this study will be useful and important resources for population genetics and phylogeographic studies. The codon type distribution was consistent (Fig 5). The codon usage bias toward a high RSCU value of U and A at the third codon position was also noted, as previously reported [43–45]. The number of RNA editing sites of Madeira *Sonchus* was significantly lower than that of *Sonchus asper* (98 sites; [20]) and mostly woody species of *Dendroseris* (93–104 sites; [42]). In general, the most frequent RNA editing sites were found in *ndh*B and *ndh*D. The Madeira *Sonchus* presented a consistent pattern also found in previous studies [46–48]. Two regions, *trn*K-*rps*16 and *ycf*1, were consistently identified as highly variable regions in the cosmopolitan weedy *Sonchus* [20] and Macaronesia endemic *Sonchus* [21]. As maternally inherited chloroplast markers, these mutation hotspots will be useful for population or phylogenetic studies of the woody *Sonchus* alliance in Macaronesia and widely distributed *Sonchus* and related species. Substantially fewer polymorphic sites were found in the Madeira *Sonchus* than in the three Canary Islands *Sonchus* species (206 polymorphic sites; [21]) and the herbaceous weedy *Sonchus* species (528 polymorphic sites; [20]). This further supports the previous view that, despite their morphological and ecological differentiation, the Madeira *Sonchus* species are highly genetically similar, suggesting their recent origin from a common ancestor most likely from the Canary Islands [12,14,49].

### Phylogenetic relationships

The phylogenetic tree shows the monophyletic clade of the woody *Sonchus* alliance in the Macaronesian Islands. Although the current study was based on a limited number of species, it is most likely that the woody *Sonchus* alliance from the Macaronesian Islands was derived from a common ancestor, as previously reported [12,14,41]. Unlike previous studies [14], the monophyly of the Madeira *Sonchus* was strongly supported in every ML, BI and MP analysis based on the complete plastome sequences. The monophyly of the Madeira clade and interspecific relationships were weakly supported based on the nrDNA ITS (65% and 71%, respectively) and few chloroplast regions (69% and 60%, respectively). One novel finding of this study is that the two conspecific subspecies of *S*. *ustulatus* are not closely related. Rather, *S*. *ustulatus* subsp. *ustulatus* shares a more recent common ancestor with *S*. *pinnatus* and *S*. *fruticosus* than with conspecific *S*. *ustulatus* subsp. *maderensis*. The previous ITS tree weakly suggested that two subspecies from coastal areas, without forming monophyly, represent basal lineages that first diverged within Madeira [14].

The plastome-based phylogenetic analysis of Madeira *Sonchus* suggests that two currently recognized subspecies of *S*. *ustulatus* may represent distinct taxonomic entities, warranting recognition at the species level. *Sonchus ustulatus* subsp. *maderensis* was proposed by Aldridge [50], based on its morphological differences and geographical distribution. *Sonchus ustulatus* subsp. *maderensis* occurs in moist rocky areas on the northern coast of Madeira (and rarely in Porto Santo and Desertas). In contrast, *S*. *ustulatus* subsp. *ustulatus* occurs in dry rocky and sunny areas on the southern coast of Madeira. These taxa are small, herbaceous perennials with acaulous or short, subwoody stems that can reach up to 30 cm in height. In contrast, *S*. *pinnatus* and *S*. *fruticose* are perennial shrubs that reach heights of up to 2 m and 4 m, respectively. Therefore, given the species distribution and chloroplast phylogenomic tree topology, it is highly likely that the common ancestor of the Madeiran *Sonchus* was somewhat similar to *S*. *ustulatus*. These were likely herbaceous perennials that first colonized the northern coastal areas of Madeira and diversified into laurel forests and moist ravines in the interior of Madeira, becoming tall shrubs. The species-level recognition of the two subspecies is further supported by a recent report of a new species, *S*. *parathalassius* J.G.Costa ex R.Jardim & M.Seq., from Porto Santo and the taxonomic recognition of *S*. *latifolia* (= *S*. *ustulauts* subsp. *maderensis*) [51]. Overall, new species-level recognition and interspecific relationships in the Madeira archipelago require further independent confirmation based on highly variable genome-wide nuclear markers.

The monophyly of the Madeira *Sonchus* was fully and strongly established in this study, but that of the closely related or progenitor *Sonchus* species from the Canary Islands remains unclear. Based on the limited Canary Islands *Sonchus* samples in this study, the plastome tree suggested that *S*. *acaulis* and *S*. *canariensis* are sisters to the Madeira clade, suggesting a possible origin from Tenerife and Gran Canaria. We gained insufficient insight into this using previous cpDNA phylogeny because of a lack of resolution within subg. *Dendrosonchus* and genus *Taeckholmia* [14]. The more resolved nuclear ITS phylogeny suggested the Madeira *Sonchus* as a sister to the clade of some Canary Islands *Sonchus* from various islands, including the older central islands (Gran Canaria, Tenerife, and La Gomera) and relatively younger western islands (La Palma and El Hierro). It makes the identification of the ancestral island and species of origin of Madeira *Sonchus* uncertain. Nevertheless, it was suggested that the origin of Madeira *Sonchus* happened relatively recently (i.e., estimated inter-archipelago dispersal event of ca. 2.7 million years ago), despite the geological age (ca. 5 million years ago) of the Madeira archipelago [49]. An extensive phylogenomic framework, including all the woody *Sonchus* alliances in the Macaronesian Islands, is required to further determine the geographical origin of the Madeira *Sonchus* species.

### Conclusion

This is the first study to report the complete chloroplast genome of four Madeira *Sonchus* species, enabling the comparison of their genomes to those of the congeneric cosmopolitan *Sonchus*, the woody Canary Islands endemic *Sonchus* from the Atlantic Ocean, and the Juan Fernández Islands endemic *Dendroseris* in the Pacific Ocean. Although the four Madeira endemic *Sonchus* species are highly morphologically and ecologically differentiated, we found nearly identical and highly conserved chloroplast genomes, supporting a single and recent origin. We identified four mutation hotspots, namely *trnK*-*rps16*, *petN*-*psbM*, *ndhF*-*Ψycf1*, and *ycf*, and 70–71 cpSSRs for population genetics, phylogeography, and phylogenetic investigation. We also characterized the codon usage bias and RNA editing sites among the four Madeira taxa and compared them with those of the Canary Islands *Sonchus* and the Juan Fernández Islands *Dendroseris*. The plastome-based phylogenetic tree strongly supported the monophyly of the Madeira lineage for the first time, but its relationship with the Canary Islands congenerics requires further study based on genome-wide nuclear and chloroplast genomes. The phylogenomic tree also suggests that the herbaceous perennial, *S*. *ustulatus* subsp. *maderensis*, which occurs on the northern coast of Madeira, was the first to diverge within the Madeira archipelago. Lastly, *S*. *ustulatus* subsp. *ustulatus*, which are herbaceous perennials occurring on the southern coast of Madeira, are more closely related to the clade of perennial shrubs, *S*. *pinnatus* and *S*. *fruticose*, than to conspecific *S*. *ustulatus* subsp. *maderensis*. This relationship corroborates the species-level recognition of *S*. *ustulatus* subsp. *maderensis* as a distinct species, *S*. *latifolia*. Further genome-wide investigations may clarify the relationship among *Sonchus* species within Madeira.

## Supporting information

S1 Table Codon usage and the codon-anticodon recognition pattern for tRNA in four Madeira *Sonchus* plastomes. (XLSX)

S2 Table Predicted RNA editing sites in the complete chloroplast genome of four Madeira *Sonchus* species. (XLSX)
